# Expression of a Mutant *kcnj2* Gene Transcript in Zebrafish

**DOI:** 10.1155/2013/324839

**Published:** 2013-11-26

**Authors:** Ivone U. S. Leong, Jonathan R. Skinner, Andrew N. Shelling, Donald R. Love

**Affiliations:** ^1^School of Biological Sciences, University of Auckland, Private Bag 92019, Auckland 1142, New Zealand; ^2^Greenlane Paediatric and Congenital Cardiac Service, Starship Children's Hospital, Private Bag 92024 Grafton, Auckland 1142, New Zealand; ^3^Department of Obstetrics and Gynaecology, University of Auckland, Private Bag 92019, Auckland 1142, New Zealand; ^4^Diagnostic Genetics, LabPlus, Auckland City Hospital, P.O. Box 110031, Auckland 1142, New Zealand

## Abstract

Long QT 7 syndrome (LQT7, also known as Andersen-Tawil syndrome) is a rare autosomal-dominant disorder that causes cardiac arrhythmias, periodic paralysis, and dysmorphic features. Mutations in the human *KCNJ2* gene, which encodes for the subunit of the potassium inwardly-rectifying channel (I_K1_), have been associated with the disorder. The majority of mutations are considered to be dominant-negative as mutant proteins interact to limit the function of wild type KCNJ2 proteins. Several LQT7 syndrome mouse models have been created that vary in the physiological similarity to the human disease. To complement the LQT7 mouse models, we investigated the usefulness of the zebrafish as an alternative model via a transient approach. Initial bioinformatic analysis identified the zebrafish orthologue of the human *KCNJ2* gene, together with a spatial expression profile that was similar to that of human. The expression of a *kcnj2-12* transcript carrying an in-frame deletion of critical amino acids identified in human studies resulted in embryos that exhibited defects in muscle development, thereby affecting movement, a decrease in jaw size, pupil-pupil distance, and signs of scoliosis. These defects correspond to some phenotypes expressed by human LQT7 patients.

## 1. Introduction

Long QT 7 syndrome (LQT7, also known as Andersen-Tawil syndrome) is a rare autosomal-dominant disorder that causes periodic paralysis, ventricular arrhythmias with QT-prolongation, and dysmorphic features, which may not be present in all affected individuals. The dysmorphic features include scoliosis (curvature of the spine), clinodactyly (permanent lateral or medial curve of a finger or toe), wide-set eyes, low set or slanted ears, small jaw, and broad forehead [1]. Mutations in the human *KCNJ2* gene, which encodes the potassium inwardly rectifying channel (I_K1_) subunit, have been associated with the disorder. The *KCNJ2* gene is expressed in the heart, brain, lung, skeletal muscle, kidney, and the eyes [2–4]. In the heart, the I_K1_ channel is involved in the resting phase of the cardiac action potential (AP) cycle.

The KCNJ2 protein consists of two transmembrane domains encompassing a selective pore region. There are currently 38 mutations reported in the Inherited Arrhythmias Database (http://www.fsm.it/cardmoc/), and 19 of these mutations have dominant-negative effects. Many of these mutations have been characterised by electrophysiological measurements of transfected CHO cells. This *in vitro* system is widely used, but for the purposes of functional studies, *in vivo* modelling is the approach-of-choice. Several LQT7 mouse models have been created and they are either *Kcnj2* knockout mutants [5, 6], or over-express wild-type (WT) Kcnj2 protein [7, 8], or the model express a dominant-negative Kcnj2 protein [8, 9].

All of these mouse models exhibit prolonged QT-interval and AP duration, as well as bradycardia (decrease in heart rate). In terms of expressing dominant-negative mutations, only one model has been described that carries a known human mutation, T75R [8], while the remaining models have expressed artificially created dominant-negative Kcnj2 proteins that do not mimic any known human mutation [9]. In general, the dominant-negative models exhibit dysmorphic features such as cleft palate that cause respiratory problems, the swelling of the stomach and small bowel [5, 6], slight narrowing of the jaw [5, 6], an increase in heart/body weight ratio [7–9], cardiac hypertrophy [7–9], and ventricular tachycardia [8]. Interestingly, over-expression of WT Kcnj2 protein causes increased mortality and cardiac hypertrophy, signs of atrioventricular block, atrial fibrillation, and premature ventricular contractions, as well as shortening of the AP duration and a consequent decrease in the QT-interval [7]. Not all the phenotypes found in the mouse models are seen in the human LQT7 condition and therefore may not be relevant.

In recent years, the zebrafish has emerged as a biomedical model for numerous human diseases, and one of these is LQT syndrome. The zebrafish genome contains a large number of orthologues of human disease-causing genes [10, 11], and these genes have been targeted to encode for proteins with similar functions to those expressed in humans. Despite the difference in anatomy, the electrophysiology of the zebrafish heart is similar to that of the human [12–16]. It has been used to model LQT2 syndrome [14, 17, 18] and in chemical screens to investigate potential drugs that could cure the cardiac arrhythmia defects caused by these mutations [19–21].

To date, there are no zebrafish models of LQT7 syndrome and no studies have been conducted in locating and verifying the zebrafish orthologue of the human *KCNJ2* gene. In this study, we have undertaken the bioinformatic identification of the zebrafish orthologue of human *KCNJ2* gene and determined the spatial and temporal expression profile of this orthologue. Wild-type *kcnj2-12* and a mutant *kcnj2-12* bearing a human LQT7 mutation were introduced into zebrafish embryos, identifying several dysmorphic features that reflect some of those seen in human LQT7 patients.

## 2. Materials and Methods

### 2.1. Bioinformatic Analysis of Zebrafish *kcnj2* Gene Orthologues

A manual reciprocal best hit approach was undertaken [22]. The human KCNJ2 amino acid sequence was queried against the zebrafish genome using the TBLASTN algorithm on Ensembl Genome Browser (Zv9 build), with search sensitivity settings on “distant homologies.” The top five results were compiled and manually annotated. A gene tree was constructed using MEGA (Molecular Evolutionary Genetics Analysis) [23] version 4.0.2. Putative zebrafish kcnj2 amino acid sequences and the mammalian and fish orthologues of the *KCNJ2*, *KCNJ4*, *KCNJ12,* and *KCNJ16* gene families were included; the corresponding accession numbers are listed in Supplementary Table 1 in the Supplementary Material available online at http://dx.doi.org/10.1155/2013/324839. Any gaps were removed from the amino acid sequence alignment and a neighbour-joining tree was constructed with 1,000 bootstrap replicates performed. The default settings were used in a *p*-distance model.

### 2.2. Zebrafish Husbandry

The wild-type (WT) zebrafish (*Danio rerio*) was maintained as previously described [24].

### 2.3. PCR and 3′ RACE

Primers were designed from putative transcript sequences and polymerase chain reaction (PCR) was performed to verify the presence of the putative zebrafish *kcnj2* orthologous genes. All primers are listed in Supplementary Table 2. PCR and 3′ RACE methods were performed as previously described [24]. The amplicons were introduced into the pGEM T-Easy vector (Promega) according to the manufacturer's instructions, and the products were bidirectionally sequenced to confirm their identity.

### 2.4. DNA Sequencing

All bidirectional DNA sequencing was carried out as previously described [25] and was undertaken by the DNA Sequencing Facility of the Centre for Genomics and Proteomics, School of Biological Sciences, The University of Auckland.

### 2.5. Tissue Collection and RNA Extraction

The following time points were collected for RNA extraction: 6 hours after fertilisation (hpf-shield), 10 hpf (bud), 14 hpf (10-somite), 16 hpf (14-somite), 19.5 hpf (21-somite), 22 hpf (26-somite), 24 hpf (prim-5), 36 hpf (prim-25), 42 hpf (high-pec), 48 hpf (long-pec), 72 hpf (protruding mouth), 84 hpf, and 96 hpf. 23–25 embryos were pooled for each time point for RNA extraction. Spatial gene expression analysis involved the following tissues: brain, gills, gut, heart, kidney, liver, muscle, and spleen. The tissues were pooled from ten adult zebrafish for RNA extraction. The RNA extractions were carried out using a combination of the Trizol (Life Technologies) and a kit-based method (Qiagen RNeasy kit) [26]. The quality of all RNA samples were analysed by an Agilent 2100 Bioanalyzer (Agilent Technologies), and only samples with an RNA integrity number (RIN) greater than 8 were used for first strand cDNA synthesis [26].

### 2.6. Quantitative Real-Time RT-PCR (qRT-PCR) and Data Analysis

All primers used for qRT-PCR are listed in Supplementary Table 2, and all primers were experimentally tested to have an amplification efficiency of more than 1.8 using the calibration dilution curve method [27]. The qRT-PCR assays were carried out as previously described [25], and three biological replicates were conducted for each temporal and spatial gene expression analysis. The raw data were inspected manually and wells with abnormal dissociation/amplification curves were discarded. The data were imported into qBase^PLUS^ software [28], and all relative gene expression calculations were carried out using this software. All gene expression was normalised against the zebrafish *ef1α* and *rpl13α* gene transcripts [24]. The geometric mean and standard error of all three biological replicates were calculated.

### 2.7. Whole Mount *In Situ* Hybridisation (WISH)

Two probes were designed to target different regions of the *kcnj2-12* gene transcript. 24 hpf and 48 hpf embryos were chosen, and WISH was performed as previously described [29].

### 2.8. *In Vitro* Site-Directed Mutagenesis

The primers used to create the WT and mutant Δ95–98 *kcnj2* recombinants are shown in Supplementary Table 2. The WT *kcnj2* was amplified using primers containing a Kozak sequence at the amino-terminus and a *Xho*I restriction enzyme site at the carboxyl-terminus. The amplicon was subsequently introduced into the pGEM T-Easy vector (Promega) according to the manufacturer's instructions, and the products were bidirectionally sequenced to confirm their identity. The QuikChange Site-Directed Mutagenesis Kit (Stratagene) protocol was used to perform *in vitro* site-directed mutagenesis to create the Δ95–98 *kcnj2* construct. The primers used in this process are shown in Supplementary Table 2.

### 2.9. *In Vitro* RNA Transcription and Expression of WT, Δ95–98 kcnj2, and Empty Control RNA Transcript

Recombinant plasmids were linearised with *Xho*I restriction enzyme and *in vitro* RNA transcription was carried out using T7 mMESSAGE mMACHINE kit (Ambion), and the transcribed mRNAs were purified using the lithium chloride precipitation method (according to the manufacturer's instructions). RNA quality was determined by gel electrophoresis and quantified using a Nanodrop ND-1000 spectrophotometer (NanoDrop Technologies, Inc.). 100 pg of Δ95–98 *kcnj2-12 *RNA, 400 pg of WT *kcnj2-12 *RNA, and 100 pg each of Δ95–98 and WT *kcnj2-12 *RNA were separately injected into one-cell stage zebrafish embryos. Empty RNA control was transcribed from plasmids that did not contain the *kcnj2-12 *gene insert.

### 2.10. Heart Rate Determination

A series of short video recordings (15 seconds) were recorded using the INFINITY 2 (Lumen*era*) CCD camera. The videos were captured using the INFINITY Capture software system and saved as. AVI files. The number of contractions was manually counted and the number of beats per minute was calculated. The average heart rate and standard error were calculated and the results were analysed using one-way ANOVA.

### 2.11. Zebrafish Motility Analysis

24 hpf dechorionated embryos were probed at the trunk of the body and yolk with fine forceps and observed under a dissecting microscope. Embryos were scored for performing a full (comprising contractions of the trunk and tail muscles) or a half-coil. The percentage of embryos performing a half-coil was determined, the standard error was calculated, and the results were analysed using one-way ANOVA to compare the differences between all samples.

### 2.12. Two Colour Acid-Free Cartilage and Bone Staining and Morphological Measurements

The cartilage and bone staining was performed as previously described [30]. The stained embryos were observed using a Zeiss Axiovert S100 microscope, and images were saved as TIFF files. The measurements were taken using ImageJ software [31].

## 3. Results and Discussion

### 3.1. Bioinformatic Analysis of Zebrafish Orthologues of the Human *KCNJ2* Gene

Several putative zebrafish orthologues of human *KCNJ2* gene can be found on the Ensembl Genome Broswer (http://www.ensembl.org/index.html); however, these sequences have not been verified and it is not known how many zebrafish *kcnj2* orthologues are present in the genome. A bioinformatic approach was undertaken to locate all possible zebrafish orthologues of the human *KCNJ2* gene. The human *KCNJ2* gene (ENST00000243457) is located on human chromosome 17 at approximately 67.10–68.10 Mb. The gene contains two exons, with only exon 2 encoding for a transcript of 1,284 bp, which is translated into a 427 amino acid residue peptide sequence (ENSP00000243457). A reciprocal best hit approach [22] was undertaken to locate all likely zebrafish orthologues of the human *KCNJ2* gene. As the query sequence was short, many results were returned from the query; however, only the five highest scoring transcripts were investigated further. These putative zebrafish orthologues were located on the following zebrafish chromosomes: chromosome 3 (*kcnj2-3* #1–3), chromosome 12 (*kcnj2-12*), and chromosome 24 (*kcnj2-24*; Supplementary Figure 1).

All five putative transcripts contained a single open reading frame; however, only the *kcnj2-12* transcript was the same length as the human *KCNJ2* gene transcript (Supplementary Table 3). The structure of the KCNJ2 protein consists of two transmembrane domains (M1 and M2) linked by a pore region. The amino- and carboxyl-termini compose the cytoplasmic domain of the ion channel and they are important for regulating the gating properties of the channel [32].

The alignment of the amino acid sequences of the putative zebrafish transcripts and the human KCNJ2 sequence is shown in Supplementary Figure 2. The kcnj2-12 sequence showed the highest sequence identity (83%) with kcnj2-3 #1, kcnj2-24, kcnj2-3 #2, and kcnj2-3 #3 showing decreasing sequence similarity to the human KCNJ2 sequence (73%, 68%, 65%, and 58%, resp.,). All five putative sequences showed at least 60% sequence identity to the critical regions M1, M2 and the pore regions of KCNJ2. According to the alignment scores, the *kcnj2-12* gene transcript was the most similar to the human *KCNJ2* gene transcript out of all five candidates; however, this was not taken to infer orthology. To determine whether *kcnj2-12* and any of the other four candidate genes were the zebrafish orthologue of the *KCNJ2* gene, a gene tree was constructed using mammalian and fish orthologues of *KCNJ2*, *KCNJ4*, *KCNJ12*, and *KCNJ14* gene families (Supplementary Figure 3). The *KCNJ* gene family all encode inward rectifier potassium channels. The *KCNJ4* gene encodes the Kv2.3 channel (expressed in heart and brain), *KCNJ12* encodes for Kv2.2 (expressed in heart and skeletal muscle), and *KCNJ14* encodes for Kv2.4 (expressed in the brain).

The gene tree shows four distinct gene families (*KCNJ2*, *KCNJ4*, *KCNJ12*, and *KCNJ14*; Supplementary Figure 3). Within each family there is a distinct mammalian and fish clade. Both *kcnj2-3* #1 and *kcnj2-12* belonged in the fish clade of the *KCNJ2* family, which suggests that the two zebrafish transcripts are paralogues and the likely coorthologues of the human *KCNJ2* gene. *kcnj2-3* #3 grouped in the fish clade of the *KCNJ4* gene family and is the apparent orthologue of *KCNJ4*. Both *kcnj2-3* #2 and *kcnj2-24* are in the fish clade of the *KCNJ12* family and are coorthologues of the human *KCNJ12* gene.

To confirm the conclusions based on the gene tree, conservation of synteny was also investigated for all five transcripts (Supplementary Figure 1). This method is complementary to the gene tree analysis and allows an inference of orthology based on the genomic context of the queried gene; the zebrafish genome exhibits extensive synteny with the human genome [33].

The human *KCNJ2* gene is flanked by the following genes: *ABCA5*, *MAP2 K6*, and *KCNJ16* (Supplementary Figure 1). The zebrafish orthologues of these genes are located on chromosome 12, with *kcnj16* and *map2k6* immediately flanking *kncj2-12* at approximately 37.40 Mb. The two genes that are adjacent to *kcnj16* are *unk* and *galr*; the human orthologues of these two genes are also located on human chromosome 17 at approximately 71.40 Mb. The zebrafish orthologue of *DNAI2*, which is a neighbouring gene of *UNK* and *GALR2*, is also located on zebrafish chromosome 12, flanked by *rpl38*, *ttyh2,* and *abca5* (this was located through the BLAST search and the transcript did not have an entry in the Zv9 database) and is located at approximately 40.40 Mb. The locations of the other four putative zebrafish transcripts are shown in Supplementary Figure 1 with the immediate flanking genes shown for each one. Only *kcnj2-3* #1 is flanked by zebrafish orthologues of *ccdc137* and *btbd17*. Based on the conservation of synteny, *kcnj2-3* #1 and *kcnj2-12* appear to be zebrafish coorthologues of *KCNJ2*, and they are paralogues of each other.

### 3.2. Transcript Analysis and Gene Expression Studies

The results from bioinformatic analysis, supported by the gene tree and the conservation of synteny, showed that *kcnj2-3 *#1 and *kcnj2-12* were the most likely zebrafish orthologues of human *KCNJ2*. These two transcripts will be further investigated while the remaining three genes were not studied further. Primers spanning the entire coding region of the *kcnj2-3 #*1 and *kcnj2-*12 transcripts were designed to confirm the sequences as well as the expression of these genes in the zebrafish heart. No PCR amplicons were obtained using the *kcnj2-3 #*1 primers, suggesting that the gene may not be expressed in the heart and therefore no further work was undertaken.

The correct sized amplicons were obtained for the *kcnj2-12* transcript, and the length and sequence matched the sequence reported in Ensembl Genome Browser (ENSDARP00000020190). The 3′ untranslated region was also identified and reported in GenBank (KC473502).

**Figure 1:**
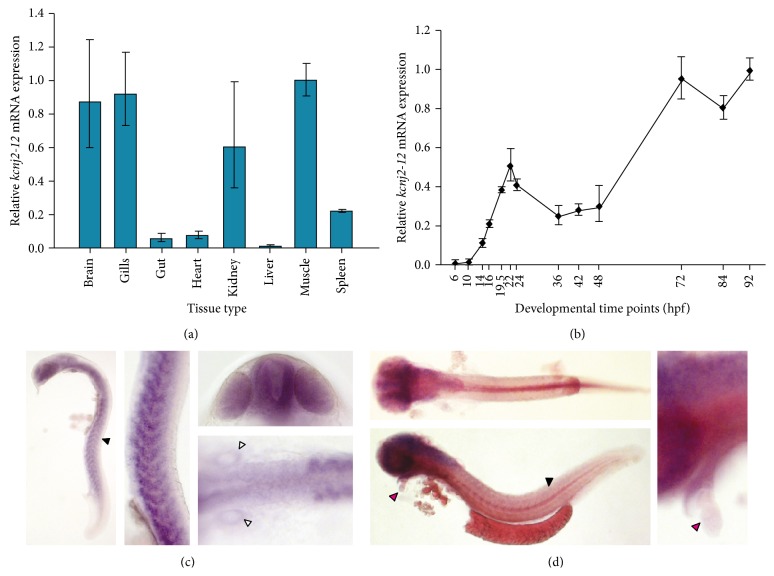
Average relative temporal and spatial gene expression and whole mount *in situ* hybridisation staining for *kcnj2-12*. (a) Average spatial gene expression profile. (b) Average temporal expression profile during embryonic development. Results for (a) and (b) were averaged across three experiments. The relative mRNA expression is quantified by qRT-PCR and all mRNA expression is relative to housekeeper genes *ef1α* and rpl13*α*. The error bars represent standard errors of the means. The tissues for each spatial gene expression profile were pooled from ten fish and 20–25 embryos were pooled for each time point of the temporal expression profile. (c) Left: side view of 24 hpf embryo showing staining throughout the whole of the body with the most staining in the brain, eyes, and somites (40x magnification; the black arrowhead indicated somite staining). Middle: a magnified side view of the defined staining in the somites (100x magnification). Right top: a magnified frontal view of the eyes and neurotube (100x magnification). Left bottom: dorsal view of staining of the otic vesicles and the somites (100x magnification; indicated by the white arrowheads). (d) Left: (top) dorsal view and (bottom) side view of 48 hpf stained embryo. Staining is strong in the brain, eyes, and slightly in the heart (40x magnification; heart staining indicated by a pink arrowhead and faint staining in the trunk indicated by a black arrowhead). Right: magnified view of the heart (100x magnification).

Spatial and temporal expression profiling of the *kcnj2-12* gene transcript was determined by quantitative real-time PCR (qRT-PCR) and whole mount *in situ* hybridisation (WISH). qRT-PCR was used to investigate the spatial expression of *kcnj2-12* in adult zebrafish and the temporal expression in young embryos. In adult zebrafish, the *kcnj2-12* gene is expressed primarily in the brain, gills, and muscle, followed by expression in the kidney. There is also some expression in the spleen. The expression in the heart and gut is low with very little or no expression in the liver (Figure 1(a)).

There was very little *kcnj2-12* gene expression before 10 hpf and there was a small rise in expression between 10 hpf and 22 hpf (Figure 1(b)); the highest level was achieved at 22 hpf. *kcnj2-12* gene expression decreased between 36 hpf and 48 hpf and then increased to its highest level after 48 hpf; peak expression was achieved at 72 hpf. The *kcnj2* expression profile may correspond to skeletal muscle development in zebrafish. At 16 hpf, somite formation is taking place. These somites will develop into myotomes that will eventually form the muscle blocks [34]. By 19 hpf, nearly all somites are chevron shaped and producing weak muscular contractions which become stronger, more coordinated, and frequent [34]. The muscle contractions form a side-to-side lashing motion at 24 hpf [34]. This series of events coincide with the peak that is seen in the *kcnj2-12* temporal profile between 10 hpf–22 hpf and could be the reason for the increase in the expression of this gene.

To investigate the spatial expression of *kcnj2-12* in young embryos, WISH was performed on embryos aged 24 hpf and 48 hpf. Two probes targeting the *kcnj2-12* transcript showed similar expression patterns. At 24 hpf, *kcnj2-12* expression was widespread, with *kcnj2-12 *expression at its highest in the brain, eyes, and somites (Figure 1(c)). The expression in the somites was well defined, but staining of the somites was not present after 48 hpf, which may be due to the RNA probe not being able to penetrate into the muscle layer at these later stages. The *kcnj2-12* gene was not expressed in the heart cone at 24 hpf; however, the heart was slightly stained at 48 hpf showing that *kcnj2-12* was expressed at low levels in this region. The expression of *kcnj2-12* in the brain and eyes were still strong at this stage (Figure 1(d)).

The human *KCNJ2* gene is expressed in the heart, brain, lung, skeletal muscle, and kidney (expression in kidney is lower than the former tissues) [3, 4] and also in the eyes [2]. The data presented here shows that spatial expression of zebrafish *kcnj2-12* is similar to the human *KCNJ2* gene spatial expression profile, with the exception that *kcnj2-12* is poorly expressed in the heart. Despite this observation, WISH staining showed that there was some cardiac expression in 48 hpf embryos, suggesting that *kcnj2-12* may only be expressed in a small region of the heart and may only take part in heart function after 24 hpf. The zebrafish *kcnj2-12* gene may play a role in muscle development in early embryos.

### 3.3. Modelling LQT7 Syndrome in Zebrafish

Of the 19 dominant-negative mutations in the *KCNJ2* gene, only two are deletion mutations and the remaining are missense mutants. The Δ95–98 mutant was chosen for further study in the zebrafish as electrophysiological studies of patients found that it has strong dominant-negative properties [35]. Over-expression of human KCNJ2 and zebrafish kcnj2-12 in zebrafish embryos have been reported by others, but not for the purposes of investigating their effects on cardiac function [36–38]. A transient zebrafish model was created to investigate the effects of expressing the zebrafish equivalent of the human Δ95–98 *KCNJ2* dominant-negative.

### 3.4. Expression of WT and Δ95–98 kcnj2-12 Proteins in Zebrafish Embryos

The WT *kcnj2-12* and the Δ95–98 *kcnj2-12* mutant were *in vitro* transcribed into RNA, and these were injected into one-cell staged zebrafish embryos. The injection of RNA was preferred over DNA in a transient zebrafish model as there is less mosaicism in transcript expression in the whole embryo. Attempts at tracking the expression of the exogenous constructs in zebrafish embryos relied on the addition of a His-tag at the aminoterminal end of each protein, and the inclusion of a downstream internal ribosomal entry site that would enable the expression of an enhanced green fluorescent protein without fusing it to the protein of interest. However, these two tracking approaches were unsuccessful and the expression of the exogenous kcnj2-12 protein could not be traced in injected zebrafish embryos.

As the expression Δ95–98 kcnj2-12 and the increased expression of WT kcnj2-12 proteins have never been studied, embryos were injected with different amounts of RNA to determine an optimum amount for subsequent experiments. Embryos were injected with 200 pg–500 pg of WT *kcnj2-12* RNA, and the proportion of dead embryos was assessed after 24 hours (Supplementary Figure 4(a)). The injection of greater than 400 pg of RNA caused morphological defects in the brain and tail region at 24 hpf (Supplementary Figure 5(a)); however, this did not affect the proportion of dead embryos (Supplementary Figure 4(a)). The midbrain-hindbrain boundary failed to develop and the head/brain region was deformed (Supplementary Figure 5(a)). The high dose of WT *kcnj2-12* RNA also affected the growth of the embryos, and injected embryos were shorter compared to control embryos. In the extremely deformed embryos, the spine was deformed and the embryos appeared shrunken with a slight hump along the spine (Supplementary Figure 5(a)). Therefore, 400 pg of WT *kcnj2-12* RNA was injected in subsequent experiments.

Unlike the WT *kcnj2-12* RNA, the injection of 400 pg of Δ95–98 *kcnj2-12* RNA caused a high proportion of embryos to die (47% compared to 19% in the group of embryos injected with empty control RNA; Supplementary Figure 4(b)). This high dose of Δ95–98 *kcnj2-12* RNA caused defects in the head/brain region, and there was no evidence of midbrain-hindbrain boundaries in these embryos. Eye development was also affected and the defects in the spine and tails were more pronounced, with some mutants exhibiting bent tails, stunted growth and protrusions along the spine (Supplementary Figure 5(b)). However, even when the amount of RNA was reduced to 150 pg there was still a large proportion of embryos dying (approximately 37%; Supplementary Figure 4(b)). At 100 pg of RNA, only 17% of embryos died, which is comparable to the embryos injected with empty control RNA (Supplementary Figure 4(b)). The aberrant features observed for higher doses of injected RNA were less severe (Supplementary Figure 5(b)), and therefore 100 pg of Δ95–98 *kcnj2-12* RNA was used for subsequent studies. These results suggest that expression of the Δ95–98 kcnj2-12 mutant was toxic to the embryos and that low levels of this mutant protein was enough to cause detrimental defects.

### 3.5. The Proportion of Dead and Aberrant Embryos

Having determined the optimum amount of Δ95–98 and WT *kcnj2-12 *RNAs to be injected into embryos, the overall effects of the introduction of these two proteins into the zebrafish were assessed based on the proportion of dead embryos after 24 hours and the identification of embryos that developed aberrant phenotypes (developmental defects in the head/brain and spine regions).

On average, 18% of embryos injected with 400 pg of WT *kcnj2-12* RNA died compared to 16% of embryos injected with an empty plasmid control (Supplementary Figure 6(a)). Coinciding with the percentage of dead embryos, the proportion of embryos exhibiting aberrant phenotypes was scored. Of the surviving embryos, only 3% exhibited an aberrant phenotype, which was exactly the same percentage as the empty control RNA embryos (Supplementary Figure 6(b)). This suggested that the zebrafish system is relatively tolerant of an increase in the WT transcript as the proportion of dead and aberrant embryos are comparable to the controls. The transgenic mouse model over-expressing WT Kcnj2 was also very tolerant to a 10-fold increase in I_K1_ density [7].

Unlike the over-expression of WT kcnj2-12 protein, 24% of embryos injected with 100 pg of Δ95–98 *kcnj2-12* RNA died compared to 16% of embryos injected with an empty plasmid control (Supplementary Figure 6(a)). This difference was not statistically significant; however, there was a significant difference between the proportion of dead embryos in the uninjected control embryos (11%) and those receiving the Δ95–98 kcnj2-12 mutant. The expression of the Δ95–98 mutant caused 31% of surviving embryos to have an aberrant phenotype, which is statistically significant compared to the empty control RNA embryos (Supplementary Figure 6(b)). Of relevance, the embryos were not tolerant of the Δ95–98 mutant for normal development. This could be taken to suggest that the endogenous kcnj2-12 protein levels are very low and that the expression of a small amount of a dominant-negative deletion mutant protein is enough to affect normal WT protein function. Four kcnj2-12 proteins are needed to assemble to create a functional I_K1_ channel [32], therefore low levels of mutant kcnj2-12 protein may be enough to compromise these channels.

To investigate whether the addition of WT kcnj2-12 protein would attenuate the effects of Δ95–98 on injected embryos, both transcripts were coinjected into one-cell staged embryos and the percentage of death and aberrant embryos were scored. Embryos receiving both the WT and Δ95–98 proteins had the lowest proportion of death (14%; Supplementary Figure 6(a)), and the percentage of aberrant embryos decreased compared to those embryos receiving only the Δ95–98 mutant transcript (Supplementary Figure 6(b)). Taken together with the proportion of dead embryos, these results suggest that expression of both WT and mutant proteins attenuated the toxicity of the deletion mutant.

The phenotypes observed in all groups of injected embryos are a result of the introduction of the WT *kcnj2-12*, Δ95–98* kcnj2-12* or a combination of both transcripts as the control embryos that have been injected with RNA that did not contain the *kncj2-12* gene did not have any adverse effects (no detectable phenotypic abnormalities).

### 3.6. Heart Rate

**Figure 2:**
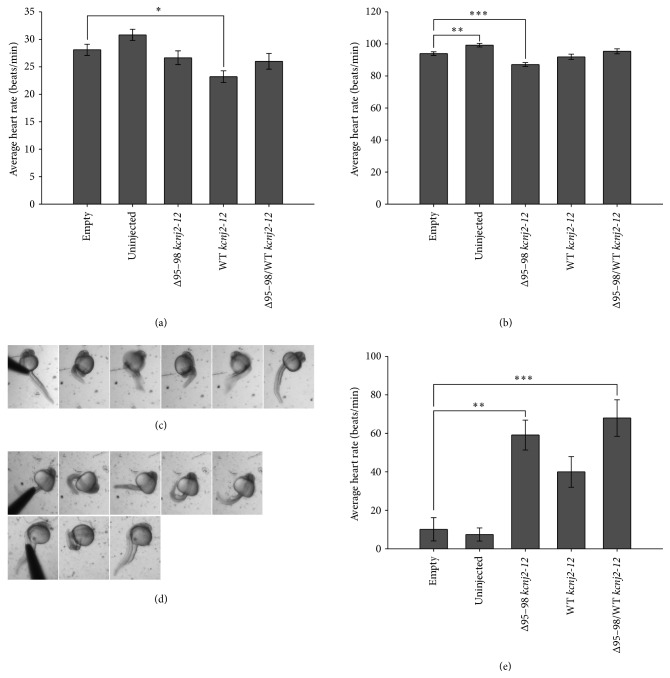
The average heart rate of embryos and the percentage of embryos unable to perform a full coil following the expression of Δ95–98 kcnj2-12, WT kcnj2-12, and combined Δ95–98 and WT kcnj2-12 proteins. (a) and (b) The average heart rate at 24 hpf and 48 hpf, respectively. (c) and (d) Representative images of embryos performing a full coil (c) and those unable to perform a full coil (d). (e) The average percentage of embryos unable to perform a full coil at 26 hpf. All results were averaged over three experiments and the error bars represent standard error for the means. The *P* values were calculated using one-way ANOVA. ^*^ *P* < 0.05, ^**^ *P* < 0.01, and ^***^ *P* < 0.001. For each experiment, each group contained ~60–70 embryos.

The heart rates of all injected embryos were assessed to determine if the expression of the Δ95–98 mutant and WT proteins had any cardiac effect. LQT7 patients show prolonged QT-intervals as a symptom of the disease, which may lead to ventricular tachycardia; however, in LQT7 mouse models, bradycardia is one of the cardiac phenotypes that was exhibited [5, 7, 9]. Therefore, the heart rates of 24 hpf and 48 hpf embryos were studied for all embryo groups (Figures 2(a) and 2(b)).

At 24 hpf, the heart rate of embryos receiving the empty control RNA was not significantly lower than the uninjected control group (28 beats/min compared to 31 beats/min; Figure 2(a)). Surprisingly, the expression of Δ95–98 protein alone and coexpression of Δ95–98 and WT proteins did not alter the heart rates of the embryos compared to control embryos (27 beats/min and 26 beats/min, resp.,). The over-expression of WT protein alone significantly lowered the heart rate to 23 beats/min compared to the empty plasmid control embryos (Figure 2(a)), which was also found in transgenic mice over-expressing WT Kcnj2 [7].

At 48 hpf, there was a statistically significant difference between the empty control RNA and uninjected control groups (94 beats/min compared to 99 beats/min, resp.; Figure 2(b)). This difference suggested that the introduction of exogenous RNA caused a reduction in heart rate, which was already observed at 24 hpf but this slowing of the heart rate became more pronounced at 48 hpf. There was a significant difference in heart rate between Δ95–98 protein alone embryos compared to empty control RNA embryos (87 beats/min compared to 94 beats/min, resp.), which is opposite to the ventricular tachycardia seen in LQT7 patients. However, this cardiac phenotype has been reported in LQT7 mouse models [5, 7, 9], suggesting that the zebrafish Δ95–98 mutant is capable of mediating a similar phenotypic outcome to the mouse models. The difference in heart rate detected at 24 hpf between the empty control RNA group and the WT-alone group was no longer present at 48 hpf (Figure 2(b)). Similar to observations made at 24 hpf, there was no difference between the heart rate of embryos coexpressing Δ95–98 and WT kcnj2-12 proteins (95 beats/min) and the empty plasmid control group.

### 3.7. Embryo Motility

In addition to cardiac features, LQT7 patients also exhibit periodic paralysis [1]. This skeletal muscle paralysis can last for hours and it is due to abnormal muscle relaxation, where muscle hyperexcitability sometimes results in inexcitability which causes abnormal relaxation of muscle [39]. After 21 hpf, normal zebrafish embryos perform a series of coilings (comprising contractions of the trunk and tail muscles) when probed with forceps at the trunk region [40, 41]. The frequency of the coilings increases over time until at 26 hpf embryos are capable of propelling themselves forward one body length [40, 41]. This response, as well as the burst swimming response (a touch stimulus applied to the tail causes the 48 hpf embryos to suddenly swim away), has been used by others to study the neuromuscular and locomotor characteristics of zebrafish [42–44].

The touch-response coiling of injected embryos at 26 hpf was investigated to determine if this was affected by the expression of the Δ95–98 and WT kcnj2-12 proteins. The zebrafish responses were separated into two categories: embryos capable of doing a full coil and those that could not (Figures 2(c) and 2(d)). When performing a full coil, the embryo contracts its tail and curls it around the base of its trunk, then it performs the coiling motion in the opposite direction before returning to its original position (Figure 2(c)). Embryos that are unable to perform a full coil still contract their tail; however, the height of the coil and contraction is not as high or as strong compared to the full coil (Figure 2(d)). There were also instances when the embryos only performed a coil in one direction and did not twist around coil in the opposite direction (Figure 2(d)).

The majority of control embryos at 26 hpf (empty control RNA and uninjected) were capable of doing a full coil when probed in the trunk area. Only 10% of the empty control RNA embryos and 7% of the uninjected control embryos were unable to perform a full coil (Figure 2(e)). Interestingly, 59% of embryos expressing Δ95–98 kcnj2-12 protein alone were unable to do a full turn, which was statistically significant compared to the empty control RNA group. This inability to perform a full coil may correspond with the Δ95–98 kcnj2-12 protein interacting in a dominant-negative manner with the WT protein and therefore causing the WT channel to not function correctly. This increased to 68% for embryos coexpressing Δ95–98 and WT kcnj2-12 proteins, which show that the additional expression of WT kcnj2-12 protein is unable to counter the effects that mutant kcnj2-12 protein has on embryo muscle contractions. Interestingly, 40% of the embryos over-expressing WT kcnj2-12 protein alone could not perform a full turn, which could be due to its role in muscle development in early embryos. Studies conducted by Yoshida et al. [38] has shown that over-expressing WT kcnj2 in zebrafish neurons can silence neuronal electrical activity and this could be what is affecting the tail coiling motion in the current study. These data suggest that coexpression of the Δ95–98 mutant and WT kcnj2-12 proteins affect embryo coiling.

**Figure 3:**
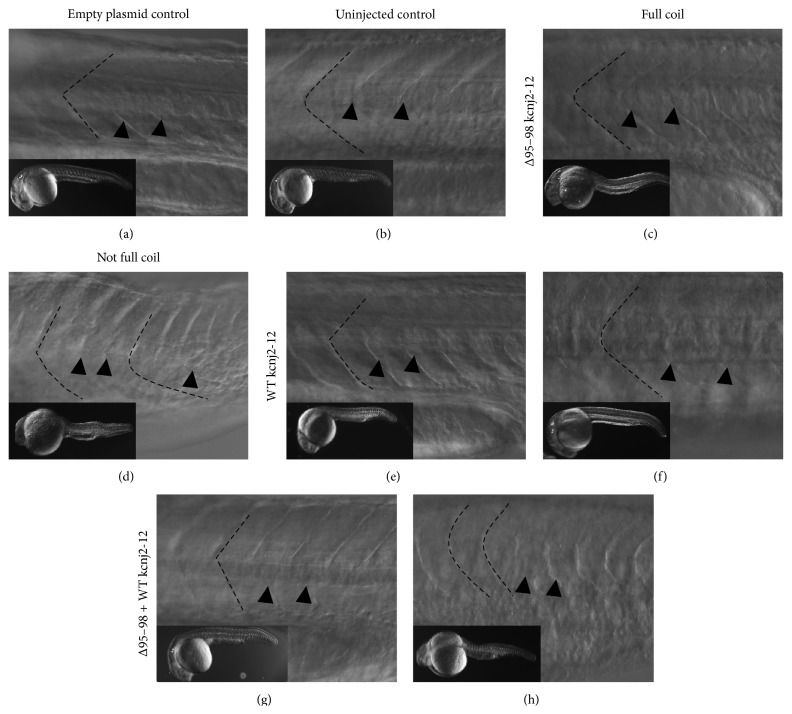
Gross morphology of somites of 26 hpf embryos expressing Δ95–98 kcnj2-12, WT kcnj2-12, and combined Δ95–98 and WT kcnj2-12 proteins under differential interference phase contrast (DIC) microscopy. (a) and (b) The somites of empty plasmid and uninjected control embryos. (c), (e) and (g) Embryos over-expressing different kcnj2-12 proteins that were able to perform a full coil. (d), (f) and (h) Embryos over-expressing different kcnj2-12 proteins that were not able to perform a full coil. The black dashed lines outline the myosepta between two somites to show the overall shape, and the black arrowheads indicate other visible myosepta boundaries.

As the motility of the embryos was affected by the coexpression of Δ95–98 and WT kcnj2-12 proteins, the gross morphology of the somites was investigated to determine if the muscles were affected. Differential interference contrast (DIC) microscopy of 26 hpf embryos was conducted. This technique enhances the contrast in unstained transparent specimens. Embryos that could not perform a full coil lost the characteristic chevron shape of the somites Figures 3(d), 3(f) and 3(h), while those that were able to perform a full turn exhibited chevron-shaped somites Figures 3(c), 3(e) and 3(g). Interestingly, the Δ95–98 mutant's somite morphology was more severely disrupted (regularity and shape). What caused this phenotype is not known. Muscle biopsy of LQT7 patients is largely avoided as a clinical diagnosis as it is based on a personal and family history of adverse cardiac events and an ECG. However, there have been a few reports of muscle biopsies taken from LQT7 patients, and those have shown tubular aggregates and very minute changes to skeletal muscle fibres [1, 35]. These observations suggest that the muscle blocks are likely to be affected in those embryos exhibiting altered motility.

### 3.8. Other Morphological Features

Patients suffering from LQT7 syndrome also display dysmorphic features including scoliosis, clinodactyly, wide-set eyes, low set, or slanted ears, which may be small or prominent, small jaw, and broad forehead [1]. To translate some of these features to the zebrafish, bone and cartilage were stained with Alizarin red and Alcian blue at 6 dpf, respectively, and the following features were measured: the width of the Meckel's cartilage, the distance between the two pupils and the width of the hyosymplectic cartilage (Supplementary Figure 7). The Meckel's cartilage forms the U-shape of the lower jaw, while the hyosymplectic cartilage supports the jaw [44].

**Figure 4:**
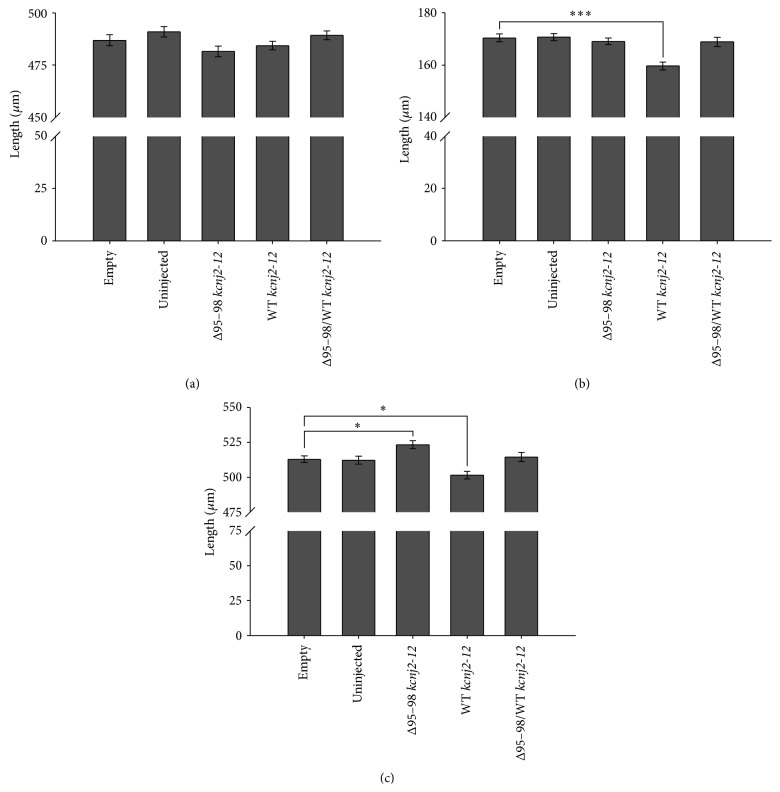
The average measurements of morphological features of 6 dpf embryos over-expressing Δ95–98 kcnj2-12, WT kcnj2-12, and combined Δ95–98 and WT kcnj2-12 proteins. (a) The average distance between the hyosymplectic cartilages. (b) The average width of the Meckel's cartilage. (c) The average pupil-to-pupil distance. The results were averaged over three experiments and the error bars represent the standard error for the means. The *P* values were calculated using one-way ANOVA. ^*^ *P* < 0.05, ^***^ *P* < 0.001.

The distance between the two hyosympletic cartilages for all embryo groups did not show a statistically significant difference (the range was from 481.5 *μ*m to 490.9 *μ*m; Figure 4(a)). There was a slight decrease for embryos over-expressing the Δ95–98 protein alone compared to the empty plasmid control (481.5 *μ*m compared to 486.8 *μ*m) and the other two groups did not show any difference at all. Despite this, the jaw sizes among embryos expressing WT kcnj2-12 protein alone were statistically different from the empty control RNA group (159.7 *μ*m compared to 170.4 *μ*m; Figure 4(b)). How the increased expression of WT kcnj2-12 protein caused this is unknown.

Unexpectedly, the embryos expressing the Δ95–98 protein, with or without WT kcnj2-12, did not show a significant difference in jaw size compared to both control groups. In the case of pupil-to-pupil distance, this was significantly longer among embryos injected with the Δ95–98 mutant compared to control embryos (523.3 *μ*m compared to 512.9 *μ*m; Figure 4(c)), while the eyes were closer together for the WT kcnj2-12 over-expressing mutants (501.5 *μ*m compared to 512.9 *μ*m). The increase in distance between the eyes for embryos injected with the Δ95–98 mutant coincided with the dysmorphic features seen in LQT7 patients. Similar to the previous two measurements, the distance between the eyes for embryos coexpressing Δ95–98 and WT kcnj2-12 proteins did not differ significantly from both control groups.

**Figure 5:**
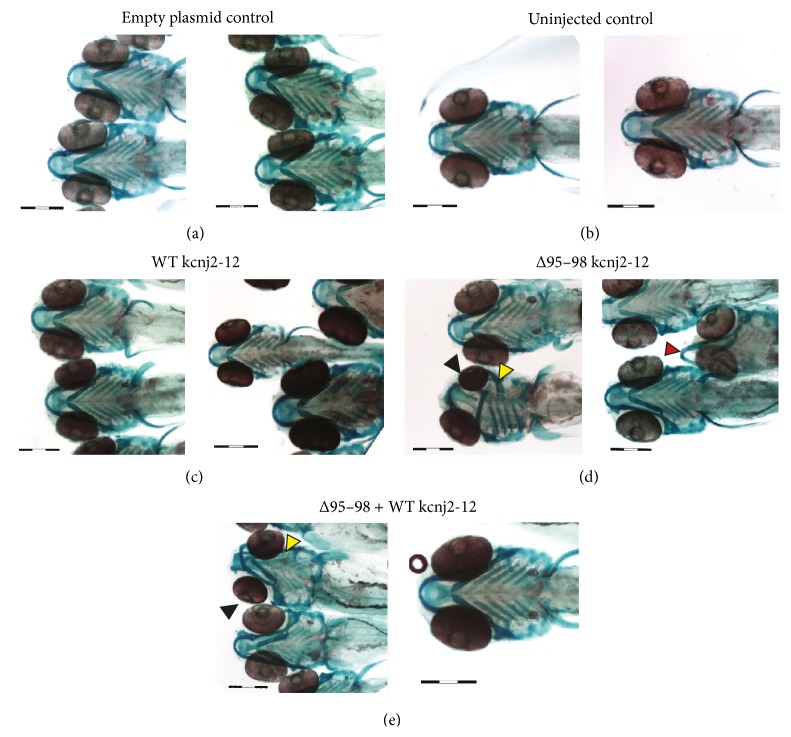
Representative images of bone and cartilage staining of 6 dpf embryos expressing Δ95–98 kcnj2-12, WT kcnj2-12, and combined Δ95–98 and WT kcnj2-12 proteins. Black arrowheads show nonuniform eye development; red arrowheads show a protruding jaw; and yellow arrowheads show deformities in the side of the jaw. Scale bars: 100 *μ*m.

The majority of the embryos appeared normal; however, the aberrant embryos in the Δ95–98 mutant alone group, and those receiving both the Δ95–98 mutant and WT kcnj2-12 that survived to 6 dpf, exhibited deformed jaws (Figure 5 and Supplementary Figure 8). For some mutants, the lower jaw protruded out and appeared beak-like, and the Meckel's cartilage remained flat and did not curl up closer to the eyes (Figure 5 and Supplementary Figure 8(b)). The formation of one side of the jaw was affected in some embryos (Figure 5 and Supplementary Figure 8). The side of the jaw (palatoquadrate and hyosympletic cartilage) and one side of the hyoid skeleton (that will become part of the gills and are made up of paired ceratohyal cartilages) were deformed. There was also evidence of eye deformities, ranging from nonuniform eye development (one eye larger than the other; Figure 5 and Supplementary Figure 8, indicated by the black arrowheads) to cyclopia (Supplementary Figure 8). As already mentioned previously, developmental defects affecting tail formation were also present (Supplementary Figure 8). Some of these appeared only as a small kink or a slight curve to the tail, while for others the tail completely curled/folded in on itself. A commonly observed mutant phenotype was the lack of recognisable body structure, except for the head and tail region (Supplementary Figure 8(a)).

Other morphological features characteristic of LQT7 is scoliosis. Like embryos injected with WT *kcnj2-12* RNA, the Δ95–98 mutants were smaller than control embryos and showed signs of abnormal spine development and tail defects.

## 4. Limitations

The low expression of the endogenous *kcnj2-12* in the zebrafish heart may suggest that the importance of this protein in the zebrafish heart may not be as great as in the human heart or it may play a different role. This would limit the use of the zebrafish as a model for LQT7 syndrome. However in this study the expression of mutant WT and Δ95–98 kcnj2-12 proteins caused the heart rate to decrease (at 24 hpf and 48 hpf, resp.), which indicates that there is a cardiac response and so this species warrants further investigation. The *kcnj2-3* #1 transcript was not further investigated because of the lack of expression in the zebrafish heart. The other transcripts identified in the bioinformatic analysis were not studied because the gene tree analysis and conservation of synteny offered very little support that these were zebrafish orthologues of the human *KCNJ2* gene.

The inability to track the protein translated from the injected RNA made it difficult to determine whether the correct protein was being created. This may be due to a low amount of RNA being injected into the embryos and therefore a low level of protein being made. The insertion of the internal ribosomal entry site between the *kcnj2-12* and the enhanced green fluorescent protein genes was to prevent the latter protein from interfering with the correct folding of the kcnj2-12 protein; however, for future studies it may be beneficial to fuse the two proteins together for easy tracking of kcnj2 protein expression in zebrafish.

The current study lacked electrophysiological profiles of the zebrafish WT and mutant kcnj2 proteins and their effect on the zebrafish cardiac system. For the present study the expression of the injected RNA would be affected by its rate of degradation and also dilution effects as cells divide and the embryos develops. This would limit the time during which electrophysiology studies could be conducted. Several electrophysiology methods could be used to study the heart in zebrafish. Two of these are electrocardiogram (ECG) and optical mapping. The earliest an ECG could be conducted in developing zebrafish is at 3 dpf [40]; however, only the P- and R-waves are detectable at this stage. In order to detect the full ECG spectrum, measurements must be taken at 7 dpf onwards [40]. For optical mapping, embryos as young as 24 hours could be studied. The technique allows the measurement of the electrical conduction throughout the whole heart and also the determination of the action potential duration of the heart [41].

Further study and histological characterisation of the muscular defects seen in the embryos would be beneficial. It would provide insight into whether the over-expression of either WT or mutant kcnj2 protein caused problems in somite/muscle development that resulted in the embryos inability to perform full coils.

## 5. Future Directions

The current study offers a possible platform to characterise LQT7 mutations found in humans and also unclassified variants in an *in vivo* model system for clinical purposes. Currently, the electrophysiology of unclassified variants are characterised in an *in vitro* system, which lacks physiological context. Therefore, creating a zebrafish equivalent of the human LQT7 mutations and assessing their characteristics in zebrafish may complement cell culture-based studies. The assessment of dysmorphic features, muscle defects, and if possible cardiac phenotypes would add value to the characterisation of unclassified variants.

For research purposes it would be beneficial to create a stable transgenic LQT7 zebrafish model. This would remove the timeframe limitation of the transient model and allow for the study of the cardiac electrophysiology of the mutants. In depth investigation into the muscle/movement defects seen in the Δ95–98 mutants could also be carried out.

Creating a stable zebrafish model containing a human LQT7 syndrome mutation would require the artificially created DNA plasmid construct to integrate into the genome. This process is time consuming with a low success rate, which can be increased by using the *Tol2* transposon system [45]. However, the gene of interest is randomly integrated into the genome and multiple copies of the gene construct may be introduced into the genome [46]. A different approach in creating a stable mutagenic line would be to use zinc-finger nucleases (ZFN), a synthetically created restriction enzyme that cleaves at targeted DNA loci [46]. In zebrafish, ZFNs introduce deletion and insertion mutations into the target site by nonhomologous end joining [46]. This event is random and subsequent DNA sequencing is needed to verify whether deletion or insertion has taken place [46]. Further investigations into the protein function would be required as the effect of the deletion/insertion mutation on the gene of interest is unknown, and the mutant protein may have a different function compared to the human mutations and may not be relevant.

The creation of a stable zebrafish LQT7 model would help with the elucidation of the aetiology of the disease and also for studying treatments for the disease.

## 6. Conclusions

The work described in this study shows that zebrafish has an orthologue of the human *KCNJ2* gene, and the spatial expression profile is similar to the human. The temporal expression profile shows that *kcnj2-12* may have a role in muscle development in zebrafish. The study also shows that LQT7 mutations could be incorporated into a WT *kcnj2-12* construct and then introduced into zebrafish embryos as a means of characterising the effects of these mutations in an *in vivo* model. The transient model was capable of reproducing some of the dysmorphic features found in LQT7 patients, which include a smaller jaw, scoliosis, and wide-set eyes. Defects in muscle development and problems with movement may also be related to the periodic paralysis found in human LQT7 patients.

Despite the limitations associated with this transient zebrafish LQT7 model, our work highlights the possibility of using this approach to characterise unclassified variants that are detected in the coding sequence of the *KCNJ2* gene in LQT7 patients. The characterisation of these mutations largely falls into two approaches: segregation analysis in the affected individual's family and *in vitro* assessment of the electrophysiological properties of the mutant protein in a cell culture system. The use of a zebrafish modelling system as described here offers a complementary approach that is worth further investigation.

## Supplementary Material

The supplementary materials contain additional figures relating to the bioinformatic analysis and expression of WT and Δ95–98 *kcnj2-12 * proteins in zebrafish embryos sections. The bioinformatic analyses include the conservation of synteny, alignment of the amino acid sequences of human KCNJ2, mouse Kcnj2 and the five putative zebrafish kcnj2 orthologues, and the unrooted gene tree for the *KCNJ2*, *KCNJ4*, *KCNJ12* and *KCNJ14* gene families. The expression of WT and Δ95–98 kcnj2-12 proteins in zebrafish embryos section include the results of the proportion of dead embryos detected 24 hours after injecting different amounts of WT *kcnj2-12* RNA and Δ95–98 kcnj2-12 RNA, the average percentage of death and percentage of deformed embryos expressing Δ95–98 *kcnj2-12*, WT kcnj2-12 and combined Δ95–98 and WT kcnj2-12 proteins, and images of aberrant embryos expressing Δ95–98 alone, and Δ95–98 and WT protein combined.
The supplemental tables include the Ensembl gene codes of all amino acid sequences used to construct the *KCNJ2* unrooted gene tree; the primer sequences used for the verification of the zebrafish orthologue of the human *KCNJ2* gene, 5' RACE of the kcnj2-12 gene transcript, qRT-PCR and construction of the *kcnj2-12 * recombinant; and the transcript and peptide lengths of all five putative zebrafish kcnj2 gene orthologues.
