# Supplementary material for: Expression of a Mutant kcnj2 Gene Transcript in Zebrafish
Source: ISRN Mol Biol. 2013 Nov 26;2013:324839. doi: 10.1155/2013/324839 (PMC4890933; doi:10.1155/2013/324839)
Supplement: Supplementary file 1 — The supplementary materials contain additional figures relating to the bioinformatic analysis and expression of WT and Δ95–98 kcnj2-12 proteins in zebrafish embryos sections. The bioinformatic analyses include the conservation of synteny, alignment of the amino acid sequences of human KCNJ2, mouse Kcnj2 and the five putative zebrafish kcnj2 orthologues, and the unrooted gene tree for the KCNJ2, KCNJ4, KCNJ12 and KCNJ14 gene families. The expression of WT and Δ95–98 kcnj2-12 proteins in zebrafish embryos section include the results of the proportion of dead embryos detected 24 hours after injecting different amounts of WT kcnj2-12 RNA and Δ95–98 kcnj2-12 RNA, the average percentage of death and percentage of deformed embryos expressing Δ95–98 kcnj2-12, WT kcnj2-12 and combined Δ95–98 and WT kcnj2-12 proteins, and images of aberrant embryos expressing Δ95–98 alone, and Δ95–98 and WT protein combined. The supplemental tables include the Ensembl gene codes of all amino acid sequences used to construct the KCNJ2 unrooted gene tree; the primer sequences used for the verification of the zebrafish orthologue of the human KCNJ2 gene, 5' RACE of the kcnj2-12 gene transcript, qRT-PCR and construction of the kcnj2-12 recombinant; and the transcript and peptide lengths of all five putative zebrafish kcnj2 gene orthologues. [file 324839.f1.zip › Supplementary material/Supplementary Material.doc]

**SUPPLEMENTARY MATERIAL**

**Supplementary Figure Legends**

**Supplementary Figure 1**

**Conservation of synteny between human *KCNJ2* and zebrafish *kcnj2-3* #1 – #3, *kcnj2-12* and *kcnj2-24* genes.**

**Upper middle panel:** The human *KCNJ2* gene (solid black) is located on chromosome 17 at approximately 67.10Mb-68.10Mb, and it is flanked by the *ABCA5*, *MAP2K6* and *KCNJ16* (dark grey). *RPL38* and *TTYH2* genes (light grey) are located at approximately 69.70Mb; *UNK*, *DNAI2*, *GALR2* and *BTBD17* genes (cross-hatch) are located at approximately 71.40Mb; and *CCDC137* (dots) is located at approximately 79.64Mb. **Upper panel:** *kcnj2-3* #1 – #3 are located on zebrafish chromosome with *kcnj2-3* #3 (solid black) located at approximately 0.50Mb, flanked by *mhclze* (white); *kcnj2-3* #1 (solid black) at 12.40Mb, flanked by *ccdc137* (dots) and *btbd17* (cross-hatch); *kcnj2-3* #2 is located at approximately 16.00Mb and it is flanked by *eftud2* and *mprip* (white). **Lower middle panel:** The *kcnj2-12* (solid black) is located on zebrafish chromosome 12 at approximately 37.40Mb, and it is flanked by *unk*, *galr2* (cross-hatch), *kcnj16* and *map2k6* (dark grey). *rpl38*, *ttyh2* (light grey), *dnai2* (cross-hatch) and *abca5* (light grey dashed box) are located at approximately 40.40Mb. **Lower panel:** The *kcnj2-24* (solid black) is located on zebrafish chromosome 24 and it is flanked by *mprip* and *dnah3* (white). White boxes represent those genes for which orthologues could not be located, and light blue dashed boxes represent genes that were located using BLAST search but the genes were not found on the Ensembl database. Dashed lines link zebrafish orthologues with the corresponding human genes. The diagram is not drawn to scale.

**Supplementary Figure 2**

**Alignment of amino acid sequences of human KCNJ2, mouse Kcnj2 and zebrafish**

**kcnj2-3 #1 (zebrafish 3 #1), kcnj2-3 #2 (zebrafish 3 #2), kcnj2-3 #3 (zebrafish 3 #3), kcnj2-12 (zebrafish 12) and kcnj2-24 (zebrafish 24).** The transmembrane domain 1 (M1) and 2 (M2), the pore region and the PDZ domain recognition sequence are marked. The similarity alignments were generated using Geneious Pro 4.8.4. Black indicates 100% conserved amino acid residues; dark grey indicates 80%-100% and light grey indicates 60%-80% conserved amino acid residues, and white indicates less than 60% conserved amino acid residues.

**Supplementary Figure 3**

**Unrooted gene tree showing the *KCNJ2*, *KCNJ4*, *KCNJ12* and *KCNJ14* gene families.**

The solid line encloses the *KCNJ2* gene family with *kcnj2-3* #1 (indicated by **, *Danio rerio* chromosome 3 #1) and *kcnj2-12* (indicated by ***, *Danio rerio* chromosome 12) grouping with the fish clade of the *KCNJ2* gene family. The *KCNJ4*, *KCNJ12* and *KCNJ14* gene families are enclosed in dashed boxes. *kcnj2-3* #2 (indicated by ††, *Danio rerio* chromosome 3 #2) and *kcnj2-24* (indicated by †, *Danio rerio* chromosome 24) group in the fish clade of *KCNJ12*, and *kcnj2-3* #3 (indicated by *, *Danio rerio* chromosome 3 #3) is in the fish clade of the *KCNJ4* family. Bootstrap values are represented as percentages.

**Supplementary Figure 4**

**Proportion of dead embryos detected 24 hours after injecting different amounts of WT *kcnj2-12* RNA (A) and Δ95-98 *kcnj2-12* RNA (B).** The results were averaged over three experiments and represented as a percentage, the error bars represent standard error for the means. For each experiment, each group contained 50 – 60 embryos.

**Supplementary Figure 5**

**Representative images of embryos 24 hours after the injection of increasing amounts of WT *kcnj2-12* RNA and Δ95-98 *kcnj2-12* RNA.** **Panel A:** Control embryos include embryos injected with empty control RNA and uninjected embryos. The images show embryos injected with 400 pg and 500 pg WT *kcnj2-12* RNA. **Panel B:** The images show embryos injected with 100 pg and 200 pg – 400 pg of Δ95-98 *kcnj2-12* RNA. The black arrowheads indicate the brain and tail abnormalities in the embryos.

**Supplementary Figure 6**

**The average percentage of death (A) and percentage of deformed (B) embryos over-expressing Δ95-98 kcnj2-12, WT kcnj2-12 and combined Δ95-98 and WT kcnj2-12 proteins.** The results were averaged across three experiments, and the error bars represent standard error for the means. The p-values were calculated using one-way ANOVA. * p < 0.05, ** < 0.01. For each experiment, each group contained ~ 130 – 140 embroys.

**Supplementary Figure 7**

**The measurements taken to assess the hyosymplectic cartilage (A), the Meckel’s**

**cartilage (B) distance between pupil-to-pupil (C).** Scale bars: 100 μm.

**Supplementary Figure 8**

**Images of different aberrant embryos expressing Δ95-98 alone (A) and Δ95-98 and**

**WT protein combined (B)**. Black arrowheads show non-uniform eye development; red arrowheads show a protruding jaw; yellow arrowheads show deformities in the side of the jaw; green arrowheads show cyclopia; and blue arrowheads show tail deformities. Scale bars: 100μm.

**Supplementary Table 1: The Ensembl gene codes of all amino acid sequences used to construct the *KCNJ2*** unrooted gene tree

| **Gene name** | **Species** | **Ensembl code** |
| --- | --- | --- |
| *KCNJ2* | *Bos taurus* | ENSBTAP00000010914 |
|  | *Canis familiaris* | ENSCAFP00000015804 |
|  | *Homo sapiens* | ENSP00000243457 |
|  | *Mus musculus* | ENSMUSP00000037192 |
|  | *Oryzias latipes* | ENSORLP00000017360 |
|  | *Pan troglodytes* | ENSPTRP00000016291 |
|  | *Rattus norvegicus* | ENSRNOP00000006254 |
|  | *Takifugu rubripes #1* | ENSTRUP00000007013 |
|  | *Takifugu rubripes #2* | ENSTRUP00000031707 |
| *KCNJ4* | *Canis familiaris* | ENSCAFP00000002016 |
|  | *Homo sapiens* | ENSP00000306497 |
|  | *Mus musculus* | ENSMUSP00000094075 |
|  | *Oryzias latipes #1* | ENSORLP00000005092 |
|  | *Oryzias latipes #2* | ENSORLP00000018318 |
|  | *Pan troglodytes* | ENSPTRP00000024751 |
|  | *Rattus norvegicus* | ENSRNOP00000053014 |
|  | *Takifugu rubripes #1* | ENSTRUP00000000108 |
|  | *Takifugu rubripes #2* | ENSTRUP00000020488 |
| *KCNJ12* | *Canis familiaris* | ENSCAFP00000027352 |
|  | *Homo sapiens* | ENSP00000328150 |
|  | *Mus musculus* | ENSMUSP00000104357 |
|  | *Oryzias latipes #1* | ENSORLP00000010256 |
|  | *Oryzias latipes #2* | ENSORLP00000014409 |
|  | *Pan troglodytes* | ENSPTRP00000053885 |
|  | *Rattus norvegicus* | ENSRNOP00000003138 |
|  | *Takifugu rubripes #1* | ENSTRUP00000021190 |
|  | *Takifugu rubripes #2* | ENSTRUP00000041874 |
| *KCNJ14* | *Bos Taurus* | ENSBTAP00000000089 |
|  | *Canis familiaris* | ENSCAFP00000005930 |
|  | *Danio rerio* | ENSDARP00000099559 |
|  | *Homo sapiens* | ENSP00000341479 |
|  | *Mus musculus* | ENSMUSP00000071829 |
|  | *Oryzias latipes* | ENSORLP00000013423 |
|  | *Pan troglodytes* | ENSPTRP00000019280 |
|  | *Takifugu rubripes* | ENSTRUP00000039730 |
|  | *Danio rerio* chromosome 3 #1 | ENSDARP00000107515 |
|  | *Danio rerio* chromosome 3 #2 | ENSDARP00000116442 |
|  | *Danio rerio* chromosome 3 #3 | ENSDARP00000089083 |
|  | *Danio rerio* chromosome 12 | ENSDARP00000020190 |
|  | *Danio rerio* chromosome 24 | ENSDARP00000078026 |

**Supplementary Table 2: Primer sequences used for the verification of zebrafish orthologue of human *KCNJ2*, 5’ RACE of *kcnj2-12*, qRT-PCR and construction of *kcnj2-12*** recombinant

| **Primer name** | **Target gene** | **Primer sequence (5’ → 3’)** |
| --- | --- | --- |
| kcnj2-12-Ex1aF | *kcnj2*-12 verification | GCACACCCGTCACCAAAC |
| kcnj2-12-Ex1aR |  | CAGCGTCTCGTTCCTTTTCT |
| kcnj2-12-Ex1bF |  | GCTTCACTGCTGCCTTTCTC |
| kcnj2-12-Ex1bR |  | AGGATTTCATTCGCCACATAG |
| kcnj2-12-Ex1cF |  | CCGCATCTTCTTGGTGTCA |
| kcnj2-12-Ex1cR |  | CCGTGCCCTTCTTCTTTCTC |
| kcnj2-3(1)-Ex1aF | *kcnj2*-3 #1 verification | AGCCCACCGATACAGCATA |
| kcnj2-3(1)-Ex1aR |  | TTCCCGACTCTCCACATTAG |
| kcnj2-3(1)-Ex1bF |  | TACTGCGGCTTTCCTGTTTT |
| kcnj2-3(1)-Ex1bR |  | TCATCCTCCGTTTCCTCTTT |
| kcnj2-24-Ex1aF | *kcnj2*-24 verification | GCGGAGGGATGAATGGATAC |
| kcnj2-24-Ex1aR |  | CACGAGAAACACAGCGAGAG |
| kcnj2-24-Ex1bF |  | GCTTGGCGTTTTGGGTTAT |
| kcnj2-24-Ex1bR |  | GCAGGATGGTGATTGGAGAG |
| kcnj2-24-Ex1cF |  | ACCGCATCTTCCTCGTCTCT |
| kcnj2-24-Ex1cR |  | TTGCTCGCTCTGGCTTTATC |
| kcnj2-12-3GSP-1F | *kcnj2*-12 3’ RACE first primer | ACCTTGCTGAGAAAAAGTACATCATATC |
| kcnj2-12-3GSP-2F | *kcnj2*-12 3’ RACE nested primer | GAACAAACACGGAAACTAGCTCAGACTC |
| kcnj2-12-rt-F | *kcnj2-12* qRT-PCR | CTGCTGTCGTGGCTGTTTT |
| kcnj2-12-rt-R |  | CCGATTGTAGTCTGCGTCTC |
| ef1á F | *ef1á* (Elongation factor á) | CTGGAGGCCAGCTCAAACAT |
| ef1á R |  | ATCAAGAAGAGTAGTACCGCTAGCATTAC |
| rpl13á F | *Rpl13á (*Ribosomal protein L13a) | TCTGGAGGACTGTAAGAGGTATGC |
| rpl13á R |  | AGACGCACAATCTTGAGAGCAG |
| kcnj2-12- Kozak-F |  | CTAGGGATCCATGGGAAGTGTGCGGGCCA |
| kcnj2-12-XhoI-R |  | CTAG**CTCGAG**TCATATTTCAGATTCCCGCC |
| kcnj2-12-Ä95-98-F | *kcnj2-12* mutagenesis primers | TTTGCTTGGCCTTCCTGCTGTTTGGATGTATCTTCTGGTT |
| kcnj2-12-Ä95-98-R | *kcnj2-12* mutagenesis primers | AACCAGAAGATACATCCAAACAGCAGGAAGGCCAAGCAAA |
|  |  |  |

The underlined sequence is the Kozak sequence; bolded sequence is the *Xho*I restriction enzyme site

**Supplementary Table 3: Transcript and peptide lengths of all five putative zebrafish *kcnj2*** orthologues

| **Gene name** | **Ensembl ID** | **Transcript length (bp)** | **Peptide length (number of residues)** | |
| --- | --- | --- | --- | --- |
| Human *KCNJ2* | ENSP00000243457 | 1,284 | 427 |  |
| ***kcnj2-3* #1** | ENSDARP00000107515 \| | 1,323 | 440 |  |
| *kcnj2-3* #2 | ENSDARP00000116442 | 1,266 | 421 |  |
| ***kcnj2-3* #3** | ENSDARP00000089083 \| | 1,317 | 439 |  |
| *kcnj2-12* | ENSDARP00000020190 | 1,284 | 427 |  |
| *kcnj2-24* | ENSDARP00000078026 | 1,314 | 437 |  |
